# Adjuvant zoledronic acid therapy for patients with early stage breast cancer: an updated systematic review and meta-analysis

**DOI:** 10.1186/1756-8722-6-80

**Published:** 2013-10-23

**Authors:** Mingfeng He, Weidong Fan, Xianquan Zhang

**Affiliations:** 1 Division of Oncology, the Second Affiliated Hospital, Chongqing Medical University, Chongqing 400010, China

**Keywords:** Bisphosphonates, Zoledronic acid, Adjuvant, Breast cancer, Meta-analysis

## Abstract

**Background:**

Zoledronic acid is a potent inhibitor of osteoclast-mediated bone resorption and has been widely used in bone metastasis malignancies and postmenopausal osteoporosis as a preventive therapy against skeletal-related events. The purpose of this study was to evaluate the clinical outcome of zoledronic acid as an adjuvant therapy for patients with early stage breast cancer.

**Patients and methods:**

Entries in the PubMed and EMBASE databases up to 12 July 2013 were systematically reviewed. Online abstracts from the proceedings of the Annual Meetings of the American Society of Clinical Oncology (ASCO) (1992–2013) and the San Antonio Breast Cancer Symposium (SABCS) (2004–2013) were also reviewed. Primary endpoints included overall survival (OS) and disease-free survival (DFS), while secondary endpoints included bone metastasis-free survival (BMFS), distant metastasis-free survival (DMFS), and fracture-free rate (FFR).

**Results:**

A total of eight studies including 3,866 subjects and 3,864 controls met our search criteria and were evaluated. The use of zoledronic acid was found to improve OS (relative risk (RR), 0.88; 95% confidence interval (CI), 0.77–1.01; *p-*value = 0.06) and DMFS (RR, 0.77; 95% CI, 0.60–1.00; *p-*value = 0.05). Furthermore, statistically significant benefits were associated with BMFS (RR, 0.81; 95% CI, 0.66–0.99; *p-*value = 0.04) and FFRs (RR, 0.75; 95% CI, 0.61–0.92; *p-*value = 0.007). In contrast, there was no significant difference in DFS with the application of zoledronic acid (RR, 0.88; 95% CI, 0.72–1.09; *p-*value = 0.24). Sensitivity analysis further identified the improvement of 5-year OS for the adjuvant zoledronic acid therapy in early stage breast cancer patients (RR, 0.86; 95% CI, 0.75–0.99; *p-*value = 0.03), while a borderline statistically significant benefit was observed for 5-year DFS (RR, 0.90; 95% CI, 0.81–1.00; *p-*value = 0.06).

**Conclusion:**

Zoledronic acid as an adjuvant therapy appears to improve the 5-year OS rate for early stage breast cancer patients, and was associated with a protective effect for the bone metastases and fractures evaluated in more than 7,000 patients. However, further research is needed to confirm our findings, and sub-group analyses according to menopause status or hormone status may provide further insight.

## Background

Breast cancer is the most commonly diagnosed malignancy and the third leading cause of cancer death among women worldwide [1]. Moreover, despite the application of neoadjuvant and adjuvant therapies to patients in the early stages of breast cancer, this disease remains a major public health challenge. In addition, since bone is the most common site for breast cancer metastasis events [2], skeletal-related events (SREs) can also develop, and these include hypercalcaemia, bone pain, pathological fractures, and spinal cord compression [3].

Bisphosphonates (BPs) are potent inhibitors of osteoclast-mediated bone resorption. Consequently, BPs have been used to treat bone metastasis malignancies [4,5] and postmenopause osteoporosis as a preventive therapy against SREs [6]. Furthermore, BPs are increasingly being used for the treatment of early stage breast cancer patients, based on level-one evidence that BPs, particularly zoledronic acid, effectively prevent cancer treatment-induced bone loss (CTIBL) in breast cancer patients receiving chemotherapy and/or estradiol-lowering endocrine therapies [7-10]. Emerging data from preclinical studies have also shown a direct anti-tumour role for zoledronic acid in the inhibition of tumour cell adhesion, invasion, and proliferation, as well as in the induction of apoptosis as demonstrated in multiple human tumour cell lines [11]. An indirect anti-tumour role for zoledronic acid also includes the inhibition of angiogenesis [12]. Considerable evidence further suggests that nitrogen-containing BPs exhibit a higher potency by exerting additive or synergistic interactions with standard cytotoxic agents [13].

Several large-scale randomized controlled trials of adjuvant zoledronic acid therapy for patients with breast cancer have recently been completed, and the results are somewhat controversial [14-17]. Therefore, a comprehensive, systematic review and meta-analysis was performed to evaluate the clinical outcome associated with the use of zoledronic acid as an adjuvant therapy for the treatment of patients with early stage breast cancer.

## Results

### Selection of studies

A total of 419 citations were obtained from electronic searches of the Pubmed and EMBASE databases. Upon review of these abstracts, 402 articles were excluded. Based on the inclusion criteria established for the present study, an additional nine articles were excluded. Thus, eight randomized controlled trials were examined [14-21], which included 7,730 patients with early stage breast cancer.

### Study characteristics

All eight studies were multicentre prospective clinical trials. The level of evidence provided by each article was graded with a Jadad quality score, and these values ranged from 2 to 5 (Table 1). Assessment of risk of bias also identified an unclear or high risk for these studies.

**Table 1:** Assessment of eligible trials according to Jadad grading

**Study**	**Random sequence generation**	**Random masking**	**Double-blind**	**Withdrawals and dropouts**	**Sum (Jadad score)**
ABCSG-12 [15]	2	2	0	1	5
AZURE [14]	2	2	0	1	5
E-ZO-FAST [20]	1	2	0	1	4
Z-FAST [17]	1	0	0	1	2
ZO-FAST [16]	1	0	0	1	2
Leal et al., [19]	1	2	0	1	4
Aft et al., [18]	1	0	1	1	3
Talahashi et al., [22]	1	0	0	1	2

Of the eight studies, the Z-FAST, ZO-FAST, and E-ZO-FAST studies [16,17,22], as well the study by Takahashi et al. [23], included hormone responsive postmenopausal women. However, while the Takahashi trial enrolled Japanese women, the other three studies primarily included Caucasian women. The ABCSG-12 trial [15] included premenopausal women with endocrine responsive breast cancers in their early stages. However, these patients were treated with goserelin for ovarian suppression, and as a result, these patients were considered postmenopausal. In the trial conducted by Leal et al. [19], postmenopausal women regardless of their hormone status were included. In the AZURE [14] and Aft et al. [18] trials, both postmenopausal and pre/perimenopausal breast cancer patients were enrolled. Furthermore, while Aft et al. represents a phase II clinical trial, the Z-FAST, ZO-FAST, E-ZO-FAST, ABCSG-12, AZURE, Takahashi et al., and Leal et al. trials were phase III clinical trials.

For two of the studies examined [20,21], data were obtained from updated study reports that were presented at the American Society of Clinical Oncology (ASCO) meetings and included a longer follow-up period (36 months). In contrast, the follow-up period of each of the other studies was approximately 5 years [14-19]. A summary of the studies included in this meta-analysis is provided in Table 2.

**Table 2:** Summary of the trials examined T

**Study year**	**Intervention**	**Bisphosphonate acid administration**	**Combination therapy**	**Duration (y)**	**No. patients per arm**	**Follow-up (months)**
ABCSG-12 (2011) [15]	Zoledronic acid Observation	4 mg IV every 6 mos	Goserelin and Tamoxifen or Anastrozole	3	900	62
					903	
AZURE (2011) [14]	Zoledronic acid Observation	4 mg IV every 4 wks × 6, then 3 mos × 8 and 6 mos × 5	Standard treatment	5	1681	59
					1678	
E-ZO-FAST (2009) [20]	Upfront zol	4 mg IV every 6 mos	Letrozol	5	263	36
	Delayed zol				264	
Z-FAST (2012) [17]	Upfront zol	4 mg IV every 6 mos	Letrozol	5	300	61
	Delayed zol				300	
ZO-FAST (2013) [16]	Upfront zol	4 mg IV every 6 mos	Letrozol	5	532	60
	Delayed zol				533	
Talahashi et al., (2013) [21]	Upfront zol	4 mg IV every 6 mos	Letrozol	1	94	36
	Delayed zol				95	
Leal et al., (2010) [19]	Zoledronic acid Observation	4 mg IV every 3 mos	Standard treatment	1	36	96
					32	
Aft et al., (2012) [18]	Zoledronic acid Observation	4 mg IV every 3 wks	Neoadjuvant chemotherapy		60	61.9
					59	

### Primary endpoints: overall survival (OS) and disease-free survival (DFS)

**Figure 1:**
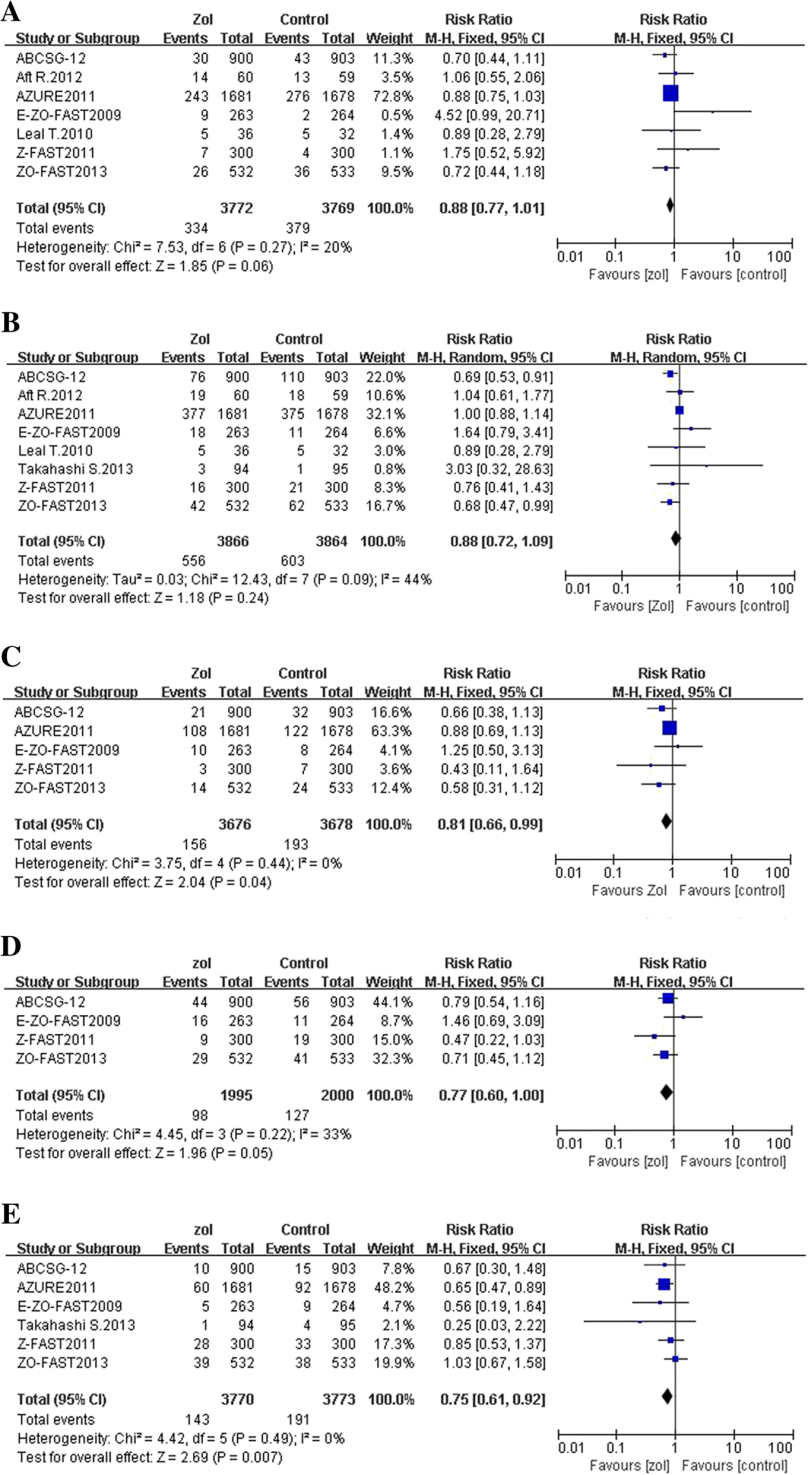
Forest plots from a meta-analysis of overall survival (A), disease-free survival (B), bone metastasis-free survival (C), distant metastasis-free survival (D), and fracture-free rate (E) for the adjuvant use of zoledronic acid compared to the control arm.

OS was assessed in seven studies (including 7,541 patients). Zoledronic acid was associated with a borderline statistically significant improvement in OS (relative risk (RR), 0.88; 95% confidence interval (CI), 0.77–1.01; *p-*value = 0.06; fixed-effect, no significant studies heterogeneity, *I*^2^ = 20%, *p-*value = 0.27) (Figure 1A). In contrast, DFS was assessed in eight studies (including 7,730 patients), and no difference in the use or absence of zoledronic acid as an adjuvant was observed (RR, 0.88; 95% CI, 0.72–1.09; *p-*value = 0.24; random-effect, significant studies heterogeneity, *I*^2^ = 44%, *p-*value = 0.09) (Figure 1B).

### Secondary endpoints: bone metastasis-free survival (BMFS), distant metastasis-free survival (DMFS), and fracture-free rate (FFR)

BMFS was reported in five studies (including 7,354 patients), and a statistically significant benefit for BMFS was identified with adjuvant zoledronic acid use (RR, 0.81; 95% CI, 0.66–0.99; *p-*value = 0.04; fixed-effect, no significant studies heterogeneity, *I*^2^ = 0%, *p-*value = 0.44) (Figure 1C). DMFS was reported in four studies (including 3,995 patients), and showed a borderline statistically significant benefit with adjuvant zoledronic acid use (RR, 0.77; 95% CI, 0.60–1.00; *p-*value = 0.05; fixed-effect, no significant studies heterogeneity, *I*^2^ = 33%, *p-*value = 0.22) (Figure 1D). Lastly, FFR was reported in six studies (including 7,543 patients), and showed a statistically significant decrease in fracture rates with zoledronic acid as an adjuvant (RR, 0.75; 95% CI, 0.61–0.92; *p-*value = 0.007; fixed-effect, no significant studies heterogeneity, *I*^2^ = 0%, *p-*value = 0.49) (Figure 1E).

### Sensitivity analysis

**Figure 2:**
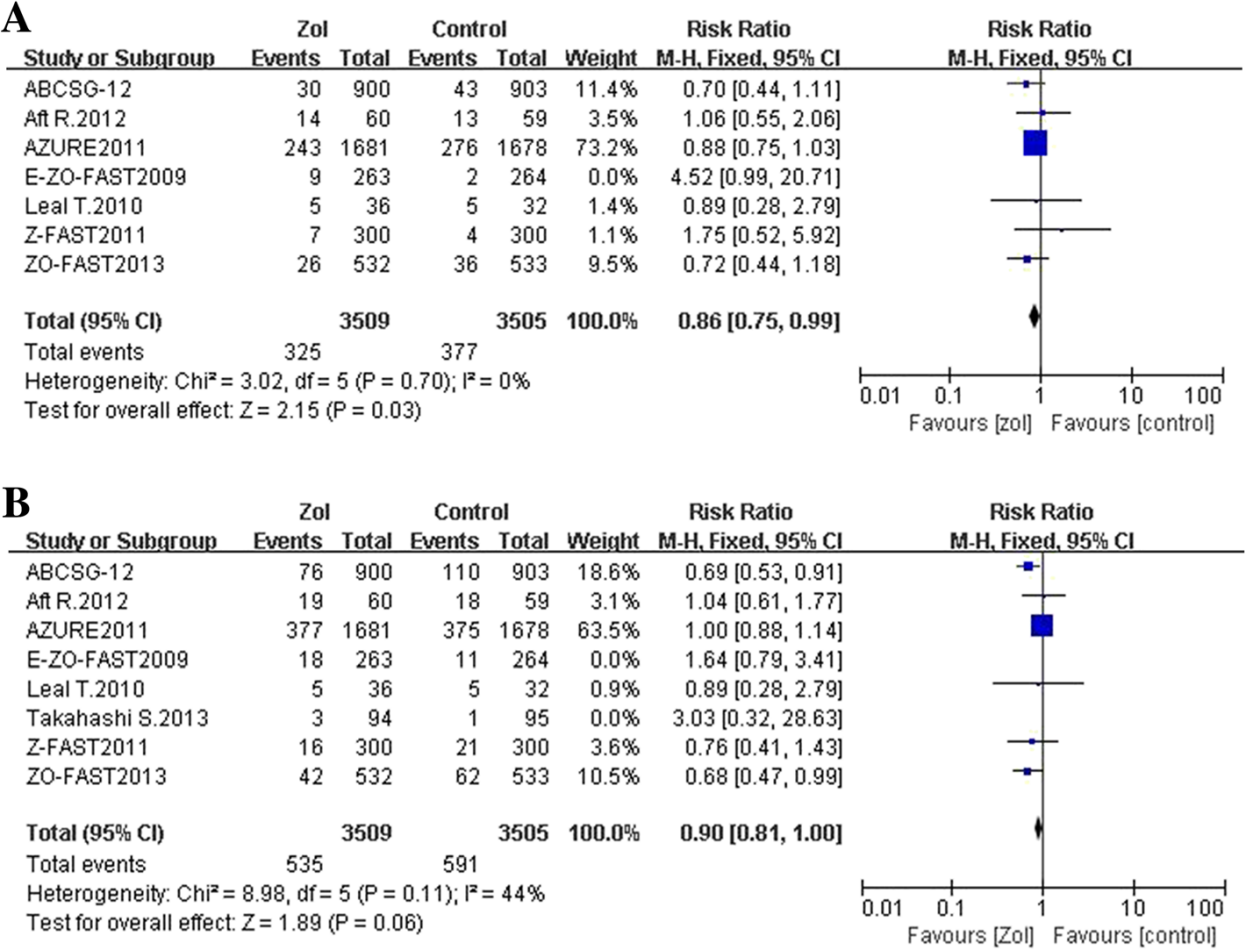
Forest plots from a sensitivity analysis of overall survival (A) and disease-free survival (B) for the adjuvant use of zoledronic acid compared to the control arm, with a median follow-up period of 5 years.

Sensitivity analysis was conducted according to the follow-up period. For two trials with a 36 month follow-up period and fewer progressive events [20,21], these were omitted for this analysis. For the six other studies, the 5-year OS rate for more than 7,000 patients in the early stages of breast cancer was found to significantly improve with zoledronic acid as an adjuvant (RR, 0.86; 95% CI, 0.75–0.99; *p-*value = 0.03; fixed-effect, no significant studies heterogeneity, *I*^2^ = 0%, *p-*value = 0.70) (Figure 2A). Moreover, an improvement trend was observed for the 5-year DFS rate for these same studies (RR, 0.90; 95% CI, 0.81–1.00; *p-*value = 0.06; fixed-effect, no significant studies heterogeneity, *I*^2^ = 44%, *p-*value = 0.11) (Figure 2B).

### Publication bias

In all of the meta-analyses performed, the funnel plots were symmetrical. These results indicate that it is unlikely that publication bias had a major influence on the analyses performed (plots not shown).

## Discussion

The meta-analysis performed included more than 7,000 patients and provides evidence that the use of zoledronic acid in an adjuvant breast cancer setting improves 5-year OS rates by 14%, reduces the risk of metastasis events by 19%, and lowers the risk of fractures by 25%. In contrast, the use of zoledronic acid did not improve DFS or DMFS, although the 5-year DFS and DMFS rates showed a borderline statistically significant benefit (the *P*-values were 0.06 and 0.05, respectively).

In a meta-analysis conducted by Zhu et al., oral clodronate was found to increase 5-year OS rates and BMFS rates [24], and our results are consistent with these outcomes. A possible explanation for the beneficial role of BPs is the direct and indirect anti-tumour activities of these compounds, which include the prevention of tumour cell adhesion to bone, the induction of tumour cell apoptosis, and an inhibition of angiogenesis [11,12]. In addition, some clinical trials have shown that zoledronic acid is able to eliminate disseminated tumour cells (DTCs) from bone marrow [25,26], thereby reducing the tumour burden in bone and preventing the development and progression of bone metastases. Zoledronic acid, as a third-generation BP, is characterized by an imidazole ring containing two nitrogen atoms, and appears to mediate stronger anti-tumour effects compared with non-nitrogen-containing BPs. For example, the presence of nitrogen has been associated with synergistic interactions with standard cytotoxic agents [13] and the regulation of T-cell-mediated host immunosurveillance of tumour cells [27]. There is also evidence that zoledronic acid may inhibit visceral metastases [28]. The Southwest Oncology Group (SWOG)-S0307 trial [29] is ongoing to compare clodronate, zoledronic acid, and ibandronate therapies in 4,500 patients with stage I–IIIa breast cancer. The goal is to establish the best choice of BPs in an adjuvant setting. In addition, the SUCCESS study [30] involving 3,700 patients in the early stages of breast cancer has been designed to compare DFS for patients undergoing chemotherapy and receiving zoledronate (4 mg) for 2 years versus 5 years. The optimal treatment time and the largest cost-benefit ratio for this cohort are also being examined. Another type of antiresorptive agent that is being evaluated is denosumab, a fully humanised monoclonal antibody that has been associated with a metastasis-prevention role and is currently being investigated in adjuvant phase III trials, including ABSCG-18 (ClinicalTrials.gov Identifier, NCT00556374) and D-CARE (ClinicalTrials.gov Identifier, NCT01077154).

In the current meta-analysis, zoledronic acid use was not found to benefit DFS and DMFS. However, the largest studies performed to date [14-16,31] have shown statistically significant benefits for the DFS and OS of postmenopausal women. Similarly, the AZURE trial [14] included a prespecified subgroup analysis based on age and menopausal status. For patients who had undergone menopause for more than 5 years before their breast cancer diagnosis, a significant improvement in DFS and OS was observed when zoledronic acid was received. In contrast, no benefit was observed for premenopausal and perimenopausal patients, or patients with unknown menopausal status, who also received zoledronic acid. In the ABCSG-12 trial [15], significant survival benefits were observed for patients > 40-years-old with low estrogen levels resulting from gonadotropin-releasing hormone use. In the final 60-month follow-up of the ZO-FAST trial [16], a statistically significant benefit for DFS (hazard ratio (HR) = 0.63; *p*-value = 0.0516) and OS (HR = 0.50; *p*-value = 0.0224) was associated with zoledronic acid use for patients who were postmenopausal for more than five years or who were > 60 years of age when starting the study. Furthermore, based on an analysis of stratification subgroups by the National Surgical Adjuvant Breast and Bowel Project protocol (B-34) [31], OS, BMFS, and non-BMFS were found to be significantly improved with clodronate treatment in women aged 50 years and older. In comparison, there was little difference observed between the clodronate group and placebo group, each containing patients younger than 50 years. In a meta-analysis conducted by Yan and Yin [32], a better DFS outcome (RR = 0.763; *p*-value < 0.001) was observed, yet a reduced risk of distant (RR = 0.744; *p*-value = 0.003) and locoregional recurrence (RR = 0.508; *p*-value = 0.001) was found for the postmenopause subgroup with zoledronic acid adjuvant use. These findings suggest that the anticancer benefits of BPs, especially zoledronic acid, have the greatest potential in a low-estrogen environment. While the underlying mechanism for this observation remains unclear, it has been hypothesized that estrogen’s effects on the bone microenvironment play a key role in determining who benefits from adjuvant BP therapy.

The results of the present study are consistent with those of the meta-analysis conducted by Valachis et al. [33]. However, there are additional considerations regarding this comparison. First, the ZO-FAST study included in the current meta-analysis included data from a 60-month follow-up period, while the Valachis study analyzed data from a 54-month follow-up period. Secondly, the ABCSG-12 trial included 62- and 84-month follow-up data, while 62-month follow-up data were used in the present study. As a result, a more reliable evaluation of 5-year OS outcome was achieved. Third, while Valachis et al. analysed the OS for only five studies, and these did not include the Z-FAST and E-ZO-FAST trials which had negative results, the present meta-analysis did include all of these studies. Correspondingly, the present study included a robust sensitivity analysis. However, our review had several limitations to consider as well. First, our analysis was based on published or presented data, and did not include individual patient data. Therefore, certain details and prespecified subgroup data could not be acquired. For example, the AZURE trial [14] did not provide distant metastases data, thereby resulting in an incomplete analysis of DMFS (p-value = 0.05) for 3,995 patients. Secondly, subgroup analysis was not performed according to menopausal status since the definition of postmenopausal status was found to vary in the articles examined. Furthermore, the majority of the subgroup analyses performed were unplanned, making comparisons unreliable. Thirdly, some of the trials considered [16,17,22,23] included a control group that received a delayed administration of zoledronic acid. Moreover, if this treatment was administered in the lumbar spine (LS) or total hip (TH), the associated T score fell below -2.0, or a non-traumatic fracture occurred. Therefore, a number of patients in the control group received zoledronic acid during follow-up, and it cannot be excluded that the effect of zoledronic acid on patient outcome was underestimated. In addition, these trials were designed to assess changes in bone mineral density (BMD) as a primary endpoint, and not DFS or OS. Fourthly, the clinical trials included in this meta-analysis utilized different methodologies, did not conduct double-blinded studies, and the schedule and duration of zoledronic acid use differed. Finally, sensitivity analysis was performed by omitting two trials having 36-month follow-up periods [22,23], and this omission affected the results obtained. It is hypothesized that the reduced number of event outcomes associated with this shorter follow-up period lacked statistical efficiency. Furthermore, the studies in the meta-analysis were found to have an unclear risk of bias or a high risk of bias, and the latter may have inflated the results obtained.

## Conclusion

In the meta-analysis performed, the use of intravenous zoledronic acid as an adjuvant therapy for patients with early stage breast cancer was found to improve 5-year OS rates and to provide a protective effect for bone metastases and fractures in more than 7,000 patients. These clinical benefits, combined with acceptable drug-specific toxicity, provide a positive risk-benefit profile for zoledronic acid as an option for adjuvant breast cancer therapy. However, as additional data becomes available, an updated meta-analysis should be performed. Furthermore, an analysis according to menopause status or hormone status should also be conducted, and this may identify an even greater benefit for zoledronic acid as an anti-tumour agent in an adjuvant setting.

## Materials and methods

### Data sources

A systematic search of the PubMed and EMBASE databases was performed of entries made through 12 July 2013. Online abstracts from the proceedings of the Annual Meetings of the American Society of Clinical Oncology (ASCO) (1992–2013) and the San Antonio Breast Cancer Symposium (SABCS) (2004–2013) were also reviewed. The terms, 'bisphosphonates’ , 'zoledronic acid’ , 'adjuvant’ , and 'breast cancer’ were used as key words.

### Study selection

A study was considered acceptable according to the following inclusion criteria: (a) it represented a prospective randomized trial, (b) the patients examined were in the early stages of breast cancer (e.g., stage I-III), (c) zoledronic acid was used in an adjuvant setting, (d) sufficient data were reported to allow an estimate of RR to be calculated for DFS and OS, and (e) English was the language of the publication. When multiple articles regarding the same trial were retrieved, the most recent or most complete publications were selected. Reports involving advanced breast cancer (e.g., recurrent breast cancer or metastatic breast cancer) were excluded. Non-randomized studies were also excluded, as well as letters to the editor, reviews, abstracts containing insufficient detail to meet the inclusion criteria, articles published in a book, and papers published in a language other than English.

### Clinical endpoints

Overall, eight studies including 3866 subjects and 3864 controls met our search criteria and were evaluated. For each of these studies, the first authors’ name, year of publication, number of patients randomly assigned, patient menopausal status, mean patient age, medication strategies for treatment and control arms, and possible outcomes at final follow-up were recorded. Primary endpoints for these studies included OS and DFS, while secondary endpoints included BMFS, DMFS, and FFR. Furthermore, the outcome measures were based on an intention-to-treat analysis. The methodological qualities of each study were evaluated using modified Jadad scoring (ranging from 0 to 7), with 0–3 indicating low quality and 4–7 indicating high quality. Cochrane Collaboration’s tool for assessing risk of bias was also employed, which includes low, unclear, and high categories of risk of bias. Any differences in assessments made by M.F.H and W.D.F were resolved through discussion.

### Statistical analysis

The Mantel-Haenszel method was used to calculate RRs and their 95% CIs using either fixed- or random-effects models, depending on the amount of heterogeneity observed. The effect of heterogeneity was quantified using *I*^2^ = 100% × (Q–df) / Q, where Q is Cochran’s χ^2^ heterogeneity statistic, df is the degrees of freedom, and *I*^2^ ranges between 0% and 100%. Moreover, *I*^2^ values of 25%, 50%, and 75% are classified as low, moderate, and high estimates, respectively [34]. Statistically significant heterogeneity was defined according to chi^2^, with a *P* value < 0.1 or an *I*^2^ value > 50%. A random-effects model meta-analysis for heterogeneous outcomes and a fixed-effect model meta-analysis for homogeneous outcomes were also performed [35]. For the sensitivity analysis performed, two studies were excluded that had follow-up periods less than five years.

Funnel plots were generated to investigate potential publication bias. All *p-*values were two-sided and a RR < 1 favored the use of zoledronic acid. All analyses were performed using Review Manager (Version 5.2. Copenhagen: The Nordic Cochrane Centre, The Cochrane Collaboration, 2012).

## Abbreviations

SRE: Skeletal-related events; BPs: Bisphosphonates; CTIBL: Cancer treatment-induced bone loss; ASCO: American Society of Clinical Oncology; SABCS: San Antonio Breast Cancer Symposium; OS: Overall survival; DFS: Disease-free survival; BMFS: Bone metastasis-free survival; DMFS: Distant metastasis-free survival; FFR: Fracture-free rate; DTCs: Disseminated tumour cells; LS: Lumbar spine; TH: Total hip; BMD: Bone mineral density; RRs: Relative risk ratios; CIs: Confidence intervals.

## Competing interests

The authors declare that they have no competing interests.

## Authors’ contributions

MFH contributed to the design of this study, literature review, data collection and analysis, and drafting of this manuscript. WDF contributed to the literature review, data collection and analysis, and critical revisions of the manuscript. XQZ contributed to the conceptualization of this study, data interpretation, and provided scientific advice. All authors have read and approved the final manuscript.

## Authors’ information

XQZ: Professor, Head of Oncology Department, the Second Affiliated Hospital, Chongqing Medical University.
